# An oncolytic adenovirus coding for a variant interleukin 2 cytokine improves response to chemotherapy through enhancement of effector lymphocyte cytotoxicity, fibroblast compartment modulation and mitotic slippage

**DOI:** 10.3389/fimmu.2023.1171083

**Published:** 2023-07-05

**Authors:** Santeri Pakola, Dafne C. A. Quixabeira, Tatiana V. Kudling, James H. A. Clubb, Susanna Grönberg-Vähä-Koskela, Saru Basnet, Elise Jirovec, Victor Arias, Lyna Haybout, Camilla Heiniö, Joao M. Santos, Victor Cervera-Carrascon, Riikka Havunen, Marjukka Anttila, Akseli Hemminki

**Affiliations:** ^1^ Cancer Gene Therapy Group, Translational Immunology Research Program, University of Helsinki, Helsinki, Finland; ^2^ TILT Biotherapeutics Ltd., Helsinki, Finland; ^3^ Helsinki University Hospital Comprehensive Cancer Center, Helsinki, Finland; ^4^ Pathology, Finnish Food Authority, Helsinki, Finland

**Keywords:** oncolytic virus, immunotherapy, chemotherapy, interleukin 2, adenovirus

## Abstract

Pancreatic ductal adenocarcinoma (PDAC) is a highly treatment-resistant cancer. Currently, the only curative treatment for PDAC is surgery, but most patients are diagnosed with metastatic disease and thus outside the scope of surgery. The majority of metastatic patients receive chemotherapy, but responses are limited. New therapeutics are thus urgently needed for PDAC. One major limitation in treating PDAC has been the highly immunosuppressive tumor microenvironment (TME) which inhibits anti-cancer immune responses. We have constructed an oncolytic adenovirus coding for a variant the interleukin 2 molecule, Ad5/3-E2F-d24-vIL2 (also known as TILT-452, and “vIL-2 virus”), with preferential binding to IL-2 receptors on the surface of effector lymphocytes over T regulatory cells (T regs). In the present study this virus was evaluated in combination with nab-paclitaxel and gemcitabine chemotherapy in Panc02 mouse model. Ad5/3-E2F-d24-vIL2 showed marked PDAC cell killing *in vitro*, alongside induction of mitotic slippage and immunogenic cell death in PDAC cell lines, when combined with chemotherapy. Increased survival was seen *in vivo* with 80% of animals surviving long term, when compared to chemotherapy alone. Moreover, combination therapy mediated enhanced tumor growth control, without observable toxicities in internal organs or external features. Survival and tumor control benefits were associated with activation of tumor infiltrating immune cells, downregulation of inhibitory signals, change in fibroblast populations in the tumors and changes in intratumoral cytokines, with increased chemokine amounts (CCL2, CCL3, CCL4) and anti-tumor cytokines (IFN-γ and TNFα). Furthermore, vIL-2 virus in combination with chemotherapy efficiently induced tumor protection upon rechallenge, that was extended to a previously non-encountered cancer cell line. In conclusion, Ad5/3-E2F-d24-vIL2 is a promising immunotherapy candidate when combined with nab-paclitaxel and gemcitabine.

## 1 Introduction

Pancreatic cancer is the seventh leading cause of cancer mortality worldwide (1). Pancreatic ductal adenocarcinoma (PDAC) is the most common form of pancreatic cancer with a 5-year survival rate of less than 10%, due to late diagnosis and high rates of treatment resistance (1, 2). Currently, the only curative treatment for PDAC is surgery in local stages of disease. However, since the majority of PDAC diagnoses are made at late stages of the disease, only a small fraction of patients benefit from surgical intervention. In metastatic disease, the prognosis is even worse, with most patients succumbing to the disease within one year after the diagnosis (2). For metastatic disease, standard care includes several chemotherapeutic options, the two most common of which are nab-paclitaxel with gemcitabine and FOLFIRINOX (folinic acid + fluorouracil + irinotecan + oxaliplatin), without superiority being demonstrated between these two approaches (2, 3). Even with chemotherapeutics, the overall survival of patients is poor, with median overall survival of 8.5 months for gemcitabine combined with nab-paclitaxel and 11.1 months for FOLFIRINOX, with higher toxicities associated with FOLFIRINOX therapy (4, 5).

Immunotherapies that have been successfully implemented in other cancers – like immune checkpoint inhibitors (ICI) for metastatic melanoma or adoptive cell therapies for B-cell leukemias – have so far been mostly ineffective in PDAC (6). The PRINCE trial investigated the combination of nivolumab with chemotherapy for PDAC with median overall survival of 16.7 months (7). However, the responses in the trial were transient, and many of the patients developed resistance and succumbed to the disease at a later timepoint (7). With regard to adaptive cell therapies, a phase 1 study utilizing mesothelin targeted chimeric antigen receptor (CARmeso) T cells studied the effects of adaptive cell therapy in 8 PDAC patients (8). The therapy showed disease stabilization in 2 patients, but with limited efficacy to the primary tumors (8). The reasons for immunotherapy resistance in PDAC are multifaceted and not completely understood yet, but one of the main recognized hallmark challenges is the strongly immunosuppressive tumor microenvironment (TME) with low amounts of effector lymphocytes, leading to diminished anti-cancer immune responses (6, 9–11).

We have previously developed an oncolytic adenovirus Ad5/3-E2F-d24-vIL2 (TILT-452, “vIL-2 virus”) encoding a variant interleukin 2 (vIL-2) cytokine with preferential stimulation of effector lymphocytes over T regs (12). This occurs due to modifications made in the protein structure leading to enhanced binding affinity to the IL-2Rβ (CD122) subunit of the IL-2αβγ trimeric receptor, with excluded need for IL-2Rα (CD25) subunit engagement to exert biological functionality. This redirects the molecule to preferentially stimulate effector lymphocytes, such as CD4+ T, CD8+ T cells and NK cells, since IL-2Rα is not required for activation of these cells, whilst T regs rely on the trimeric receptor (13). As a monotherapy, the vIL-2 virus showed the ability to efficiently reprogram the tumor microenvironment and to elicit anti-tumor responses in hamster PDAC tumors (12).

## 2 Materials and methods

### 2.1 Cell lines

Human PDAC cell lines PANC-1 and Capan-2, and murine T cell line CTLL-2 and colorectal adenocarcinoma cell line MC-38 were purchased from American Type Culture Collection (ATCC) (Manassas, USA). Human PDAC cell lines BxPC-3, MIA PaCa-2 and HPAF-II were a kind gift from Adjunct Professor Hanna Seppänen from the University of Helsinki. Murine PDAC cell line Panc02 was a kind gift from Dr. Kayoko Hosaka, Karolinska Institute. All cell lines were cultured under recommended culture conditions, and cultures were passaged three to four times prior to experimental use.

### 2.2 Virus constructs

Ad5/3-E2F-d24-vIL2 construction has been previously described (12). Briefly, Ad5/3-E2F-d24-vIL2 is a serotype 5 adenovirus with knob replacement from adenovirus serotype 3. Cancer cell specific replication is achieved through addition of E2F promoter and 24kb deletion in the E1A gene. Gene for vIL-2 cytokine is inserted to the E3 region of the genome. All viruses used in the experiment utilize the Ad5/3-E2F-d24 backbone structure described previously (14). Viral construct coding for human IL-2 have also been described previously (12, 14).

### 2.3 Virus and chemotherapy cytotoxicity, transgene production and functionality

Ad5/3-E2F-d24-vIL2 (vIL-2 virus) cytotoxic capability was evaluated in different cell lines with the MTS assay. Briefly, cancer cells were plated in 96-well plates in triplicates at 1x10^4^ cells/well for 24 hours and infected with different multiplicities of infection, of either Ad5/3-E2F-d24 backbone virus or Ad5/3-E2F-d24-vIL2. After 5 days of incubation, cell viability was evaluated by incubating the cells for 2 hours with 10% CellTiter 96 Aqueous One Solution (Promega, WI, USA). Absorbance was read at 490 nanometers using Hidex Sense plate reader (Hidex, Turku, Finland). Data was normalized to uninfected control group values. For *in vitro* combination studies with chemotherapies, Paclitaxel (T7402, Sigma-Aldrich Merck, MA, USA) and gemcitabine (G6423, Sigma-Aldrich Merck, MA, USA) were prepared under sterile conditions. Cell cycle effects of paclitaxel were evaluated after 48 hour incubation utilizing a protocol described elsewhere (15).

Virus transgene production in different cell lines was evaluated with the human IL-2 Flex Set (BD Biosciences, CA, USA) according to manufacturer’s instructions. Sample IL-2 amounts were acquired with Accuri C6 Flow cytometer (BD Biosciences, CA, USA) and analyzed with FCAP Array software (BD Biosciences, CA, USA).

Virus transgene functionality was assessed using a protocol described previously utilizing Panc-1 cells (14).

### 2.4 Evaluation of immunogenic cell death

Immunogenic cell death was measured by extracellular ATP (ATP Determination Kit, A22066, Thermo Fisher Scientific, MA, USA) and calreticulin expression (Calreticulin Polyclonal Antibody, PA3-900, Thermo Fisher Scientific, MA, USA). Panc-1 cells were seeded to 12-well plates at 10^5^ cells per well and incubated with vIL-2 virus (100 VP/cell) at 100 VP/cell, gemcitabine and paclitaxel. After 48 hours, supernatants were used for ATP measurements. For calreticulin evaluation, cells were dissociated from the plates after 48 hours, and stained with primary and secondary antibodies (Donkey Anti-Rabbit IgG (H+L) conjugated to Alexa Fluor 488, Thermo Fisher Scientific, MA, USA) for flow cytometric analysis.

### 2.5 Animal studies

Immunocompetent 5-week-old female C57BL/6J (Envigo, IN, USA) mice were used for pre-clinical *in vivo* studies. Mice were engrafted subcutaneously in the lower left flank with 1x10^6^ Panc02 cells. After tumor size reached 3 to 4 mm (day 4 post engraftment) animals were randomized to different treatment groups (n=13 or 14). Virus treatments (3x10^9^ VP) were given intratumorally every two days from day 0 to 50. Chemotherapy as 50 mg/kg of nab-paclitaxel (Abraxane, Celgene Bristol Myers Squibb, NY, USA) and 100 mg/kg of gemcitabine (Accord Healthcare, UK) was given intraperitoneally every seven days from day 0 to 50. Tumor size was measured with digital calipers, and tumor volume was calculated by the formula of (length x width^2^)/2. Normalized tumor growth curves were generated by normalizing tumor volumes to Day 0. Animal weight was followed to monitor for therapy related adverse events.

On day 10, 5 animals per group were euthanized for “mechanism of action” studies, after receiving a total of 2 rounds of chemotherapy treatment and 6 rounds of virotherapy. Tumors and spleens were collected for mechanistic analyses. Parts of hearts, lungs, livers, spleens and kidneys were collected for toxicity evaluation. Remaining animals (n=8 or 9 animals per group) received therapy until day 50, when the treatment was ceased, and continued for survival studies. Animals were euthanized whenever maximum tumor diameter (18 mm) was reached or animal well-being was compromised.

Animals that showed complete tumor regression on day 150 day, were rechallenged on the upper left and right flank with the original cancer cell line and with previously non-encountered colorectal cancer cell line MC-38. Tumor development was followed for 31 days after which all animals were euthanized, and spleens and tumors were collected for further analysis.

### 2.6 Flow cytometric analysis

Tumors and spleens collected on day 10 of the animal experiment were processed with a tissue grinder and passed through a 70 µM filter to produce a single cell suspension for flow cytometric analysis. Additionally, red blood cells from collected spleens were lysed with ACK buffer (Thermo Fischer Scientific, MA, USA). A list of antibodies used can be found in **Supplementary Table 1**. Samples were analyzed with Novocyte Quanteon Flow Cytometer (Agilent Technologies, CA, USA). 30 000 to 100 000 events were acquired for each sample. Acquired flow cytometry data files were analyzed with FlowJo software 10.8.1 (BD Biosciences, CA, USA). Unstained, single stained and fluorescence-minus-one controls were used for accurate gating.

### 2.7 Histopathological analysis for therapy associated toxicity

Selected organs collected on day 10 (hearts, lungs, livers, spleens, and kidneys) were embedded in 10% formalin for 24 hours, after which samples were transferred to 70% ethanol. Samples were processed into paraffin blocks, sectioned to slides and analyzed by a veterinary pathologist in a blind manner.

### 2.8 Cytokine and chemokine analysis of tumors

Tumor fragments were snap frozen upon collection on day 10 and stored in -80°C for further analysis. Tumor proteomic analysis was carried out with Legendplex Mouse Cytokine Release Syndrome kit (Biolegend, CA, USA) according to manufacturer’s instructions. Protein concentration was normalized to the total protein content in the samples.

### 2.9 Statistical analysis and data presentation

Tumor growth curves were analyzed with mixed-model analysis utilizing logarithmic transformed normalized tumor volumes in SPSS v.29 (IBM, IL, USA). R-Studio v.4.2.2 (RStudio, MA, USA) was used to analyze survival curves by weighted log-rank test. For other analyses, GraphPad Prism v.9.2.0. (GraphPad Software, CA, USA) was used for graphical presentation and statistical analysis. Changes in re-challenge tumor rejection were calculated by Fisher’s exact test. Unpaired t-test with Welch’s correction was used to test for statistical significance between groups. Results were considered statistically significant if the p-value was less than 0.05. BioRender was used for graphical illustration.

### 2.10 Ethical statement

All animal experiments and procedures were approved by Animal Experimentation Board of the Provincial Government of Southern Finland (license number ESAVI/12559/2021) and conducted following Federation of European Laboratory Animal Science Associations (FELASA) guidelines.

## 3 Results

### 3.1 Ad5/3-E2F-d24-vIL2 efficiently lyses a multitude of different human PDAC cell lines, produces a functional cytokine and synergizes with chemotherapy

**Figure 1:**
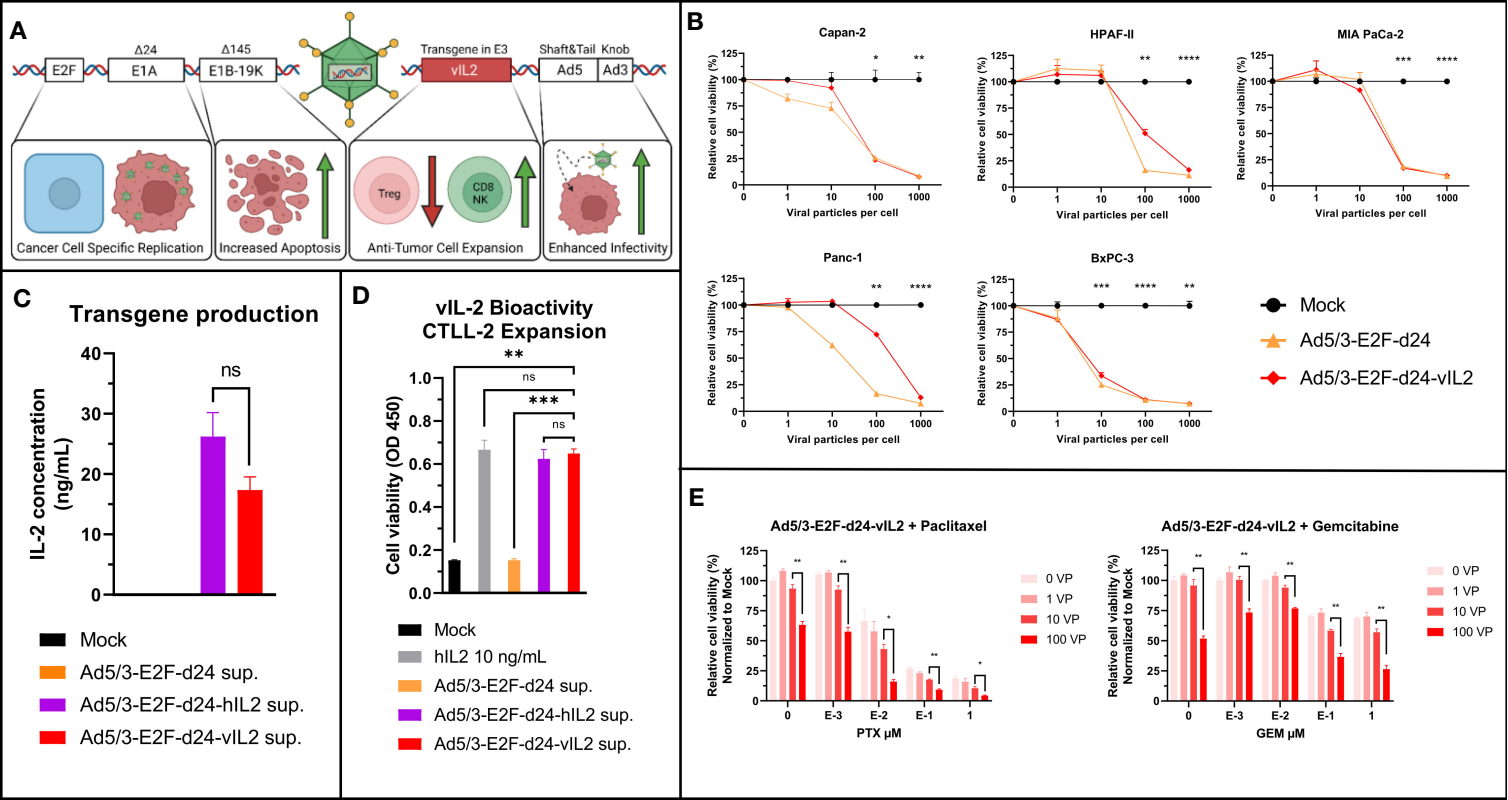
Ad5/3-E2F-d24-vIL2 (TILT-452) viral structure and *in vitro* testing. **(A)** Graphical illustration of Ad5/3-d24-E2F-vIL2 genetic structure. **(B)** Human commercial PDAC cell lines used for testing the oncolytic ability of Ad5/3-E2F-d24-vIL2. Relative cell viability by MTS and statistical difference to Mock on day 5 shown. **(C)** Transgene production in supernatants from Panc-1 cells infected with unarmed, human IL2 and variant IL2 coding viruses for 48 hours. **(D)** Variant IL2 bioactivity assessed by CTLL-2 expansion following supernatant or human recombinant IL2 administration. **(E)** *In vitro* evaluation of combination of Ad5/3-d24-E2F-vIL2 in combination with paclitaxel and gemcitabine in Panc-1 cells 5 days post treatment. Difference between 100 VP group and all other groups calculated by unpaired t-test with Welch’s correction. Significance between 10 and 100 VP shown. Error presented as +/- SEM. Significance denoted as p-value < 0.05 = *, < 0.01 = **, < 0.001 = ***, <0.0001 = ****. N=3 biological replicates for all experiments. ns = not significant.

A detailed structure of Ad5/3-E2F-d24-vIL2 has been described before (12). A graphical presentation of the viral construct can be seen in **Figure 1A**. Ad5/3-E2F-d24-vIL2 was able to infect and lyse different human PDAC cell lines with similar efficacy when compared to an unarmed virus (**Figure 1B**). The ability of the virus to produce a functional transgene was evaluated by measurement of IL-2 from supernatants (**Figure 1C**) 48 hours post infection, where Ad5/3-E2F-d24-vIL2 produced similar levels of transgene as virus encoding the normal human IL-2. Functionality of the transgene product was evaluated by CTLL-2 expansion (**Figure 1D**). Supernatant from Ad5/3-E2F-d24-vIL2 infected cells was able cause similar cell expansion when compared to virus with the standard human IL-2, and human recombinant IL-2 protein.

The viability of cancer cells after the treatment with of vIL-2 virus in combination with standard pancreatic cancer chemotherapeutics, gemcitabine and paclitaxel, was evaluated. vIL2 virus cell killing was not inhibited by the chemotherapeutic agents, and the addition of virotherapy to chemotherapy increased cell killing in all tested settings (**Figure 1E**). Even with high concentrations of paclitaxel and gemcitabine, addition of virus led to an increase in cell killing. Overall, Ad5/3-E2F-d24-vIL2 can efficiently lyse human PDAC cells, produce a functional cytokine and synergize with standard chemotherapy of PDAC.

### 3.2 Ad5/3-E2F-d24-vIL2 synergizes with paclitaxel therapy by driving cells from mitotic arrest to mitotic slippage and apoptosis

**Figure 2:**
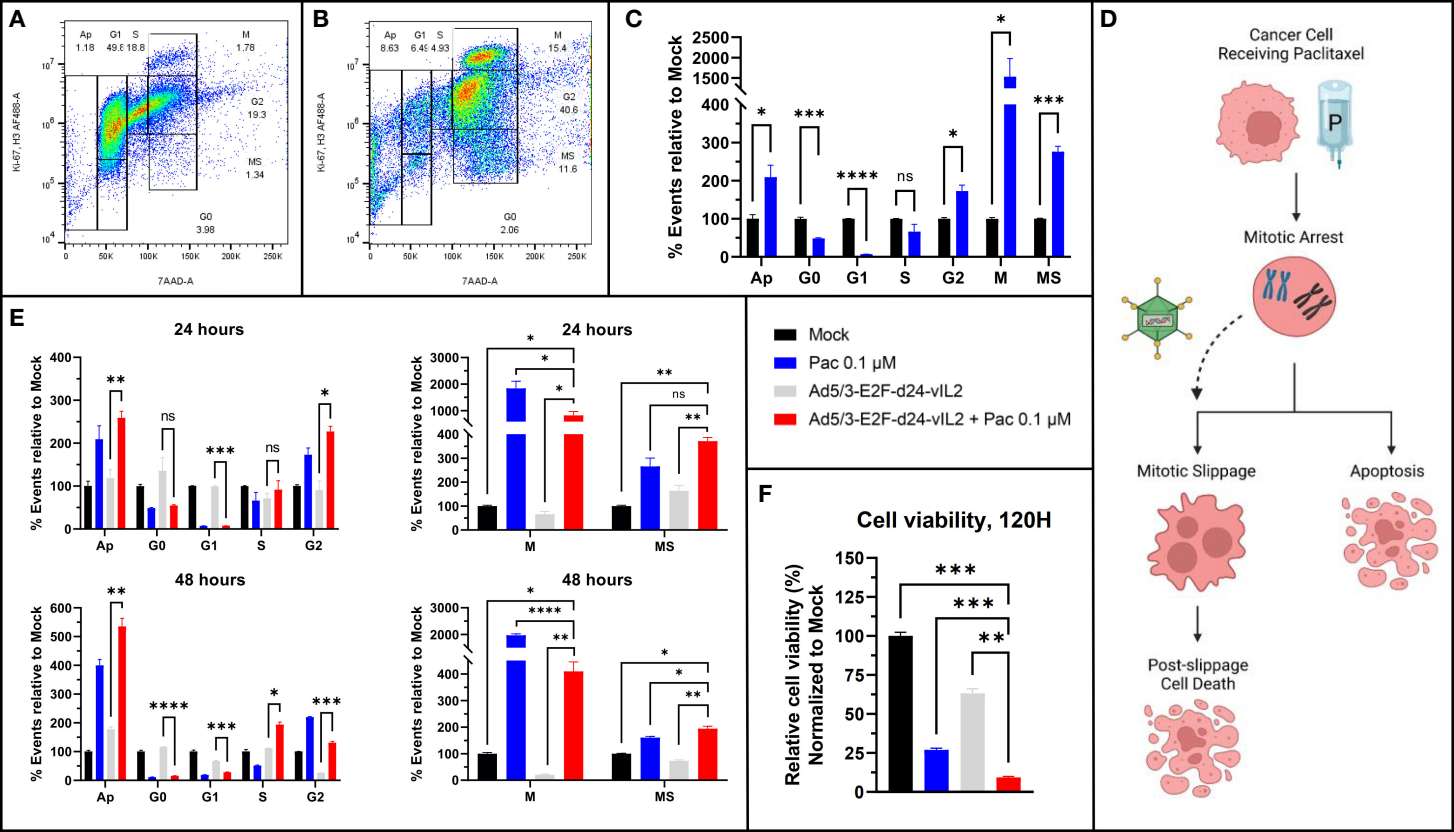
Cell cycle analysis. **(A)** Untreated Panc-1 cells and **(B)** Panc-1 cells treated with paclitaxel after 48 hours. **(C)** Changes in cell cycle population proportions after paclitaxel treatment, showing G2 and M-stasis. **(D)** Graphical illustration of proposed mechanism of action. **(E)** Cell population changes after 24 and 48 hours of treatment with paclitaxel, Ad5/3-E2F-d24-vIL2 and combination of Ad5/3-E2F-d24-vIL2 to paclitaxel. Cell proportions normalized to untreated cells. **(F)** Cell viability after 120 hours after treatment. N=3. Difference between groups measured by an unpaired T-test with Welch’s correction. Error presented as SEM. Significance denoted as p-value < 0.05 = *, < 0.01 = **, < 0.001 = ***, <0.0001 = ****.

Synergism of Ad5/3-E2F-d24-vIL2 with paclitaxel, a microtubule stabilizing chemotherapeutic agent, was further evaluated utilizing a flow cytometric protocol developed to characterize all phases of cell cycle (15). The complete flow cytometric gating strategy is shown in **Supplementary Figure 1**. First, we aimed to confirm the usability of the protocol in solid cancer cells, since the original protocol was developed for blood cancer cells. Indeed, when Panc-1 cells were left untreated (**Figure 2A**) or treated with paclitaxel (**Figure 2B**), the expected cells distribution was observed with predicted G2- and M-phase arrest typical for microtubule stabilizing agents (p=0.011 and p=0.033 respectively) compared to mock treated Panc-1 cells (**Figure 2C**). Histogram plots of aforementioned cells are shown in **Supplementary Figure 2**.

When studying the combination treatment of paclitaxel and virus, we identified increasing amounts of cells with high 7AAD binding, alongside with low binding of H3Ser10 and Ki-67 antibodies (“MS”, **Figure 2B**). Thus, these cells had a high DNA content reminiscent of mitotic and G2 cells, but low mitotic activity, reminiscent of cells in mitotic slippage (16). We then evaluated the cell cycle status after virotherapy, chemotherapy and combination treatment 24 hours and 48 hours after treatment (**Figure 2E**). When comparing cell proportions in mitosis and mitotic slippage, we saw a statistically significant shift in cell population from mitosis to mitotic slippage when paclitaxel treatment was combined with Ad5/3-E2F-d24-vIL2 (p=0.0016 and p=0.032 respectively). We thus hypothesize, that combination of Ad5/3-E2F-d24-vIL2 to paclitaxel treatment can drive cells from mitotic arrest to mitotic slippage.

To evaluate if this leads to higher cancer cell killing, we evaluated viability of Panc-1 cells 120 hours after treatment (**Figure 2F**). The addition of Ad5/3-E2F-d24-vIL2 to paclitaxel treatment was able to induce statistically higher cell killing (p=0.00050) when compared to all other groups. Thus, we suggest that the combination of vIL-2 virus with paclitaxel therapy shifts cells from M-phase stasis to mitotic slippage, and further into post-slippage cell death at a later timepoint. A graphical representation of the suggested mechanism is shown in **Figure 2D**.

### 3.3 Ad5/3-E2F-d24-vIL2 in combination with gemcitabine and paclitaxel enhances immunogenic cell death

**Figure 3:**
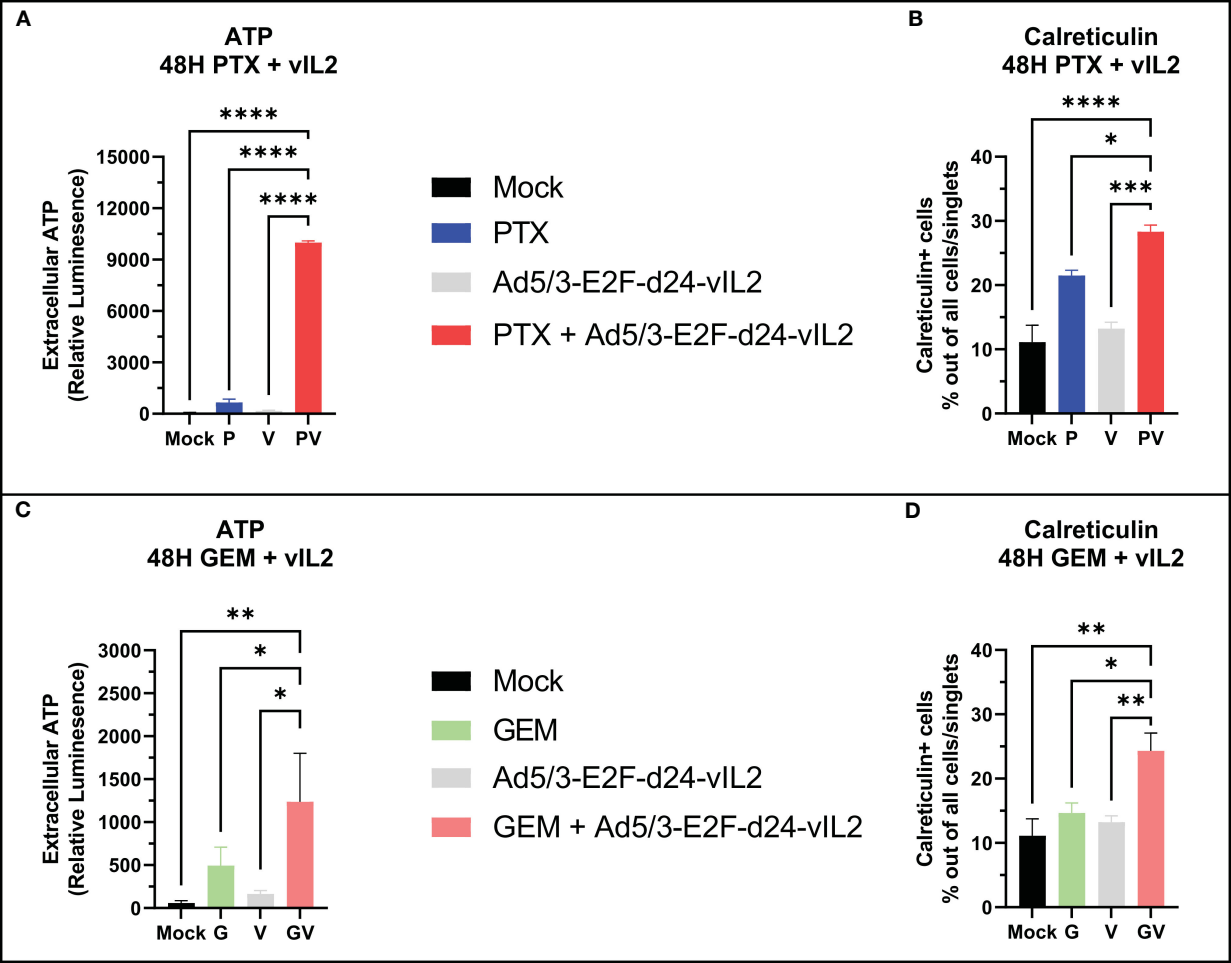
*In vitro* immunogenic cell death evaluation. **(A)** Extracellular ATP release and **(B)** calreticulin expression from Panc-1 cells when vIL-2 virus is combined with paclitaxel. **(C)** Extracellular ATP release and **(D)** calreticulin expression from Panc-1 cells when vIL-2 virus is combined with gemcitabine. Difference between groups measured by an unpaired T-test with Welch’s correction. Error presented as SEM. Significance denoted as p-value < 0.05 = *, < 0.01 = **, < 0.001 = ***, <0.0001 = ****. N=3 biological replicates for all experiments.

Next, we aimed to characterize the nature of cell death by the combination of Ad5/3-E2F-d24-vIL2 with standard chemotherapeutic agents for PDAC, specifically gemcitabine and paclitaxel. We treated Panc-1 cells with gemcitabine and paclitaxel and evaluated markers of immunogenic cell death 48 hours later. When comparing extracellular ATP amounts, the combinations of both chemotherapies to Ad5/3-E2F-d24-vIL2 were able to produce significantly (p<0.0001 for paclitaxel, p=0.039 for gemcitabine) higher expression of extracellular ATP (**Figures 3A, B**) compared to chemotherapy only. Interestingly, the combination with paclitaxel was able to produce markedly high extracellular ATP amounts compared to gemcitabine. Next, we quantified the amount of calreticulin positive cells in the cultures. Similarly, the combination of both chemotherapies was able to induce statistically higher (p=0.0079 for paclitaxel, p=0.013 for gemcitabine) amounts of calreticulin positive cells when compared to chemotherapy only (**Figures 3C, D**).

### 3.4 Ad5/3-E2F-d24-vIL2 synergizes *in vivo* with current standard of care chemotherapy in Panc02 mouse model

**Figure 4:**
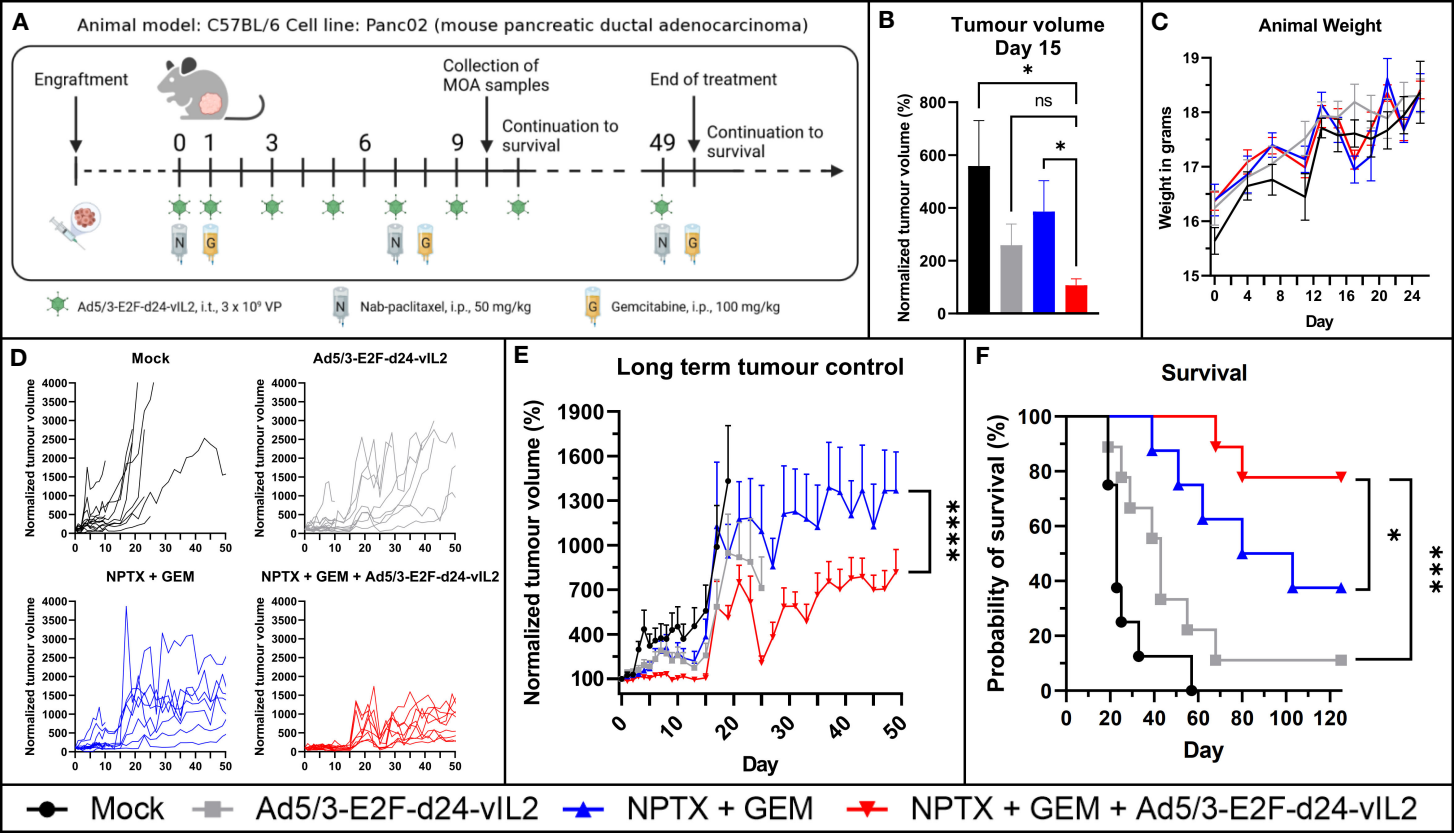
*In vivo* evaluation of Ad5/3-E2F-d24-vIL2 in combination with nab-paclitaxel and gemcitabine in Panc02 mouse model. **(A)** Animal experiment plan and treatment schedule. **(B)** Short term tumor control. **(C)** Animal weight changes during the therapy. Difference measured with unpaired t-test with Welch’s correction. **(D)** Individual tumor growth curves. **(E)** Long term tumor growth curves. Group plotted until 70% of the animals in the group are alive. Difference measured with linear mix-model analysis. **(F)** Kapplan-Meier survival plot of the animals. N=8 or 9 per group. Difference measured with weighted log-rank test. Error presented as SEM. Significance denoted as p-value < 0.05 = *, <0.001 = ***, <0.0001 = ****. ns = not significant.

Next, we aimed to assess the *in vivo* efficacy of combination treatment (**Figure 4A**). Human oncolytic adenoviruses replicate poorly in mouse cells (17, 18), thus we studied our viruses’ replicative ability in the mouse PDAC cell line Panc02 before engraftment. As expected, the virus was not able to lyse the cells even at late time points (**Supplementary Figure 1A**). However, when evaluating cytokine production, we observed that the cell line was able to produce transgene product in a dose-dependent manner, with 10-fold increase when virus concentration was proportionally increased (**Supplementary Figure 1B**). Thus, this model lacks the effects of oncolysis on tumor control, but it incorporates to some extent the effects of transgene production including effects on mouse lymphocytes. However, because of the former, this model underestimates the overall anti-tumor effects of the proposed therapy.

The combination of Ad5/3-E2F-d24-vIL2 with nab-paclitaxel and gemcitabine showed a statistically significant (p=0.049) tumor growth control advantage already at an early timepoint when compared to chemotherapy alone (**Figure 4B**). Animal weight was followed for safety evaluation, and no statistically significant weight changes between the groups were observed (**Figure 4C**). Individual tumor growth curves are shown in **Figure 4D**. When evaluating long term tumor control, the combination of Ad5/3-E2F-d24-vIL2 with chemotherapy proved to significantly (p<0.0001) enhance anti-tumor response (**Figure 4E**) when compared to all other groups. Additionally, the combination treatment was able to provide a statistically significant (p=0.042 *vs.* chemotherapy, p=0.00071 *vs.* virotherapy, p<0.0001 *vs.* mock) survival benefit compared to all other groups (**Figure 4F**), with 80% of animals surviving in the combination treatment group.

No signs of toxicity associated with the therapies was observed at histopathological analysis of collected internal organs (**Supplementary Table 2**), and no toxicity visible by animal external features or behavior were observed, in line with previously published results (19, 20).

### 3.5 Ad5/3-E2F-d24-vIL2 treatment increases intratumoral effector lymphocyte proportions and cytotoxicity in treated tumors

**Figure 5:**
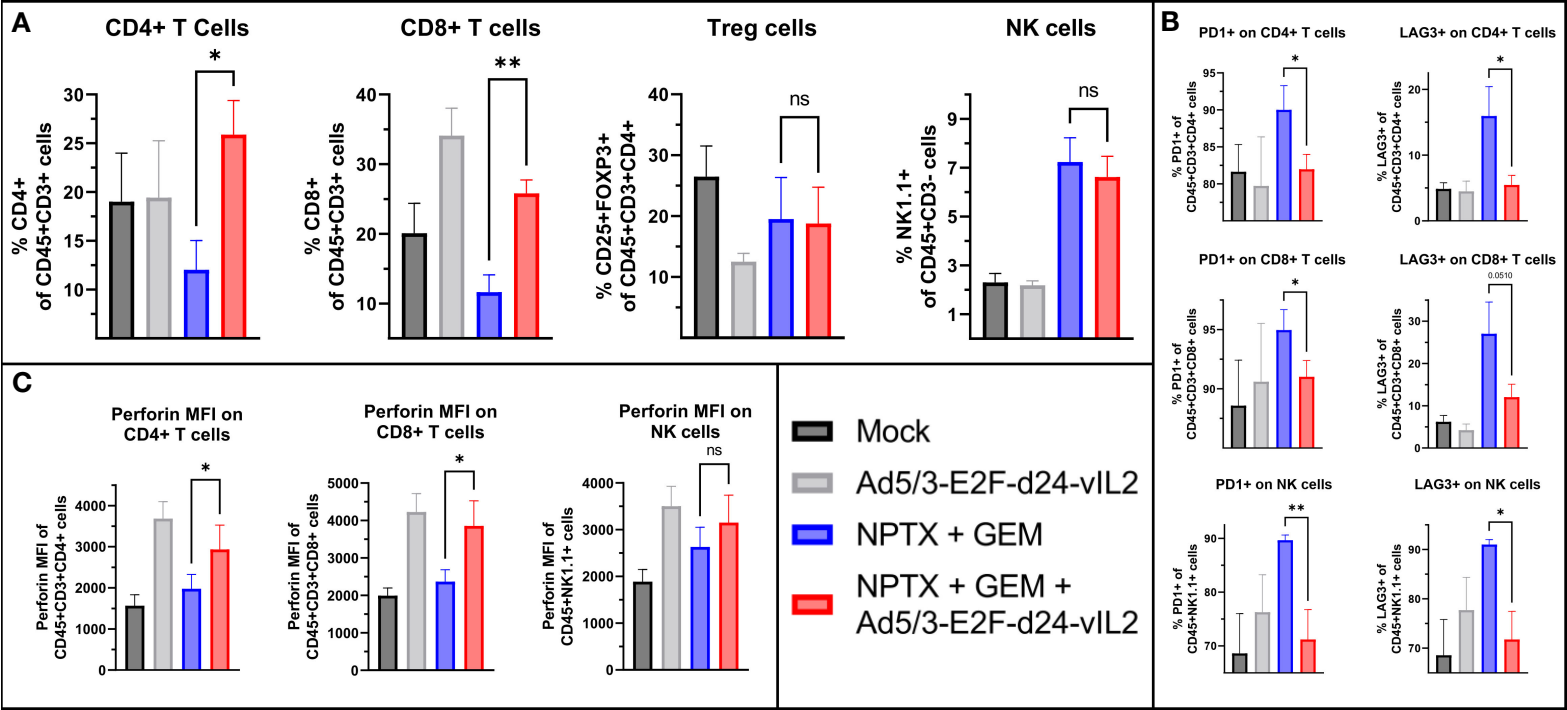
Flow cytometric analysis of tumors collected on day 10. **(A)** Changes in intratumoral CD4+, CD8+ T cells and NK cells between the groups. **(B)** Changes in intratumoral PD1+ and LAG3+ CD4+, CD8+ T cells and NK cells. **(C)** Changes in perforin surface density of intratumoral CD4+, CD8+ T and NK cells. Difference between groups measured by an unpaired T-test with Welch’s correction. Error presented as SEM. Significance denoted as p-value < 0.05 = *, < 0.01 = **. N=5 biological replicates for all experiments.

We evaluated tumor immune cell compartments by flow cytometry from samples collected on day 10. When comparing the combination of Ad5/3-E2F-d24-vIL2 with chemotherapy to chemotherapy alone, we observed a higher percentage of CD4+ and CD8+ T cells (p=0.018 and p=0.0031, respectively), with no change in NK cells or Tregs (**Figure 5A**). Next, we assessed inhibitory markers PD1 and LAG3 on immune cell subsets. CD4+ T and CD8+ T cells as well as NK cells in the chemotherapy group showed highest proportion of PD1+ and LAG3+ cells (**Figure 5B**). The combination of vIL-2 to chemotherapy did not cause upregulation of these markers when compared to chemotherapy alone. We also evaluated the functional state of these cells by perforin expression (**Figure 5C**). CD4+ and CD8+ T cells, but not NK cells in the combination treatment showed significantly higher expression of perforin (p=0.048, p=0.046, p=0.26 respectively) when compared to chemotherapy alone.

**Figure 6:**
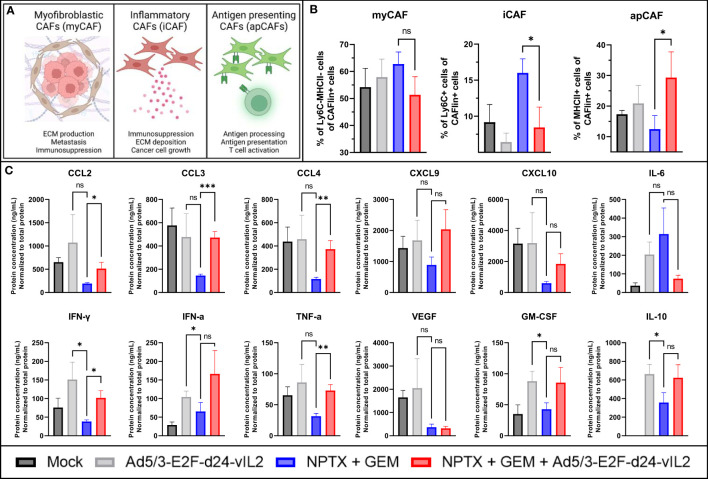
*In vivo* evaluation of TME on day 10. **(A)** Graphical representation of different CAFs and their functions. **(B)** Evaluation of intratumoral CAF subsets by flow cytometry. CAFlin = CD45-EPCAM-PDPN+ **(C)** Changes in intratumoral cytokines. Difference between groups measured by an unpaired T-test with Welch’s correction. Error presented as SEM. Significance denoted as p-value < 0.05 = *, < 0.01 = **, < 0.001 = ***. N=5 biological replicates for all experiments.

Dense stroma inhibits the efficacy of many treatments through compression of blood vessels and physical barriers (21, 22). Thus we aimed to study cancer associated fibroblasts (CAFs) found in the collected tumors (23). We evaluated the three major subsets of CAFs: myofibroblastic CAFs (myCAFs), inflammatory CAFs (iCAFs) and antigen presenting CAFs (apCAFs) (23). Major functions of these subtypes are presented in **Figure 6A**. When evaluating these cell subsets by flow cytometry, no changes in myCAFs were seen between the treatment groups (**Figure 6B**). When comparing iCAFs, a statistically significant decrease (p=0.033) in iCAFs was seen in groups receiving the combination treatment when compared to chemotherapy group. When comparing the proportions on apCAFs, a statistically significant (p=0.048) increase in apCAFs was observed in the combination therapy group. Cell population changes normalized to tumor volume on Day 10 are presented in **Supplementary Table 3**.

We also evaluated the cytokine and chemokine expression profile in tumors collected on day 10 (**Figure 6C**). When comparing the chemotherapy monotherapy group to the combination therapy group, we saw statistically significant increases in chemokines CCL2, CCL3 and CCL4 (p=0.041, p<0.0001 and p=0.0068, respectively), and a trending increase in CXCL9 and CXCL10 expression. Decrease in IL-6 and increase in IL-10 was also noted, but not significant. Statistically significant upregulation in IFN-γ and TNF-α (p=0.022 and 0.0017, respectively) was seen, with a trend in upregulation for IFN-α. No change in VEGF was seen, and a trending increase in GM-CSF was noted. Regarding virus monotherapy, vIL-2 alone was also able to produce similar changes in the cytokine and chemokine profile, as the virus combined to chemotherapy, with higher expression of CXCL10, IFN-γ and VEGF. Taken together, this cytokine data shows marked TME re-engineering elicited by the combination of Ad5/3-E2F-d24-vIL2 to nab-paclitaxel and gemcitabine chemotherapy regimen in Panc02 mouse model, resulting in anti-tumor effects.

### 3.6 Ad5/3-E2F-d24-vIL2 in combination with chemotherapy results in protection from tumor re-challenge and new tumor challenge

**Figure 7:**
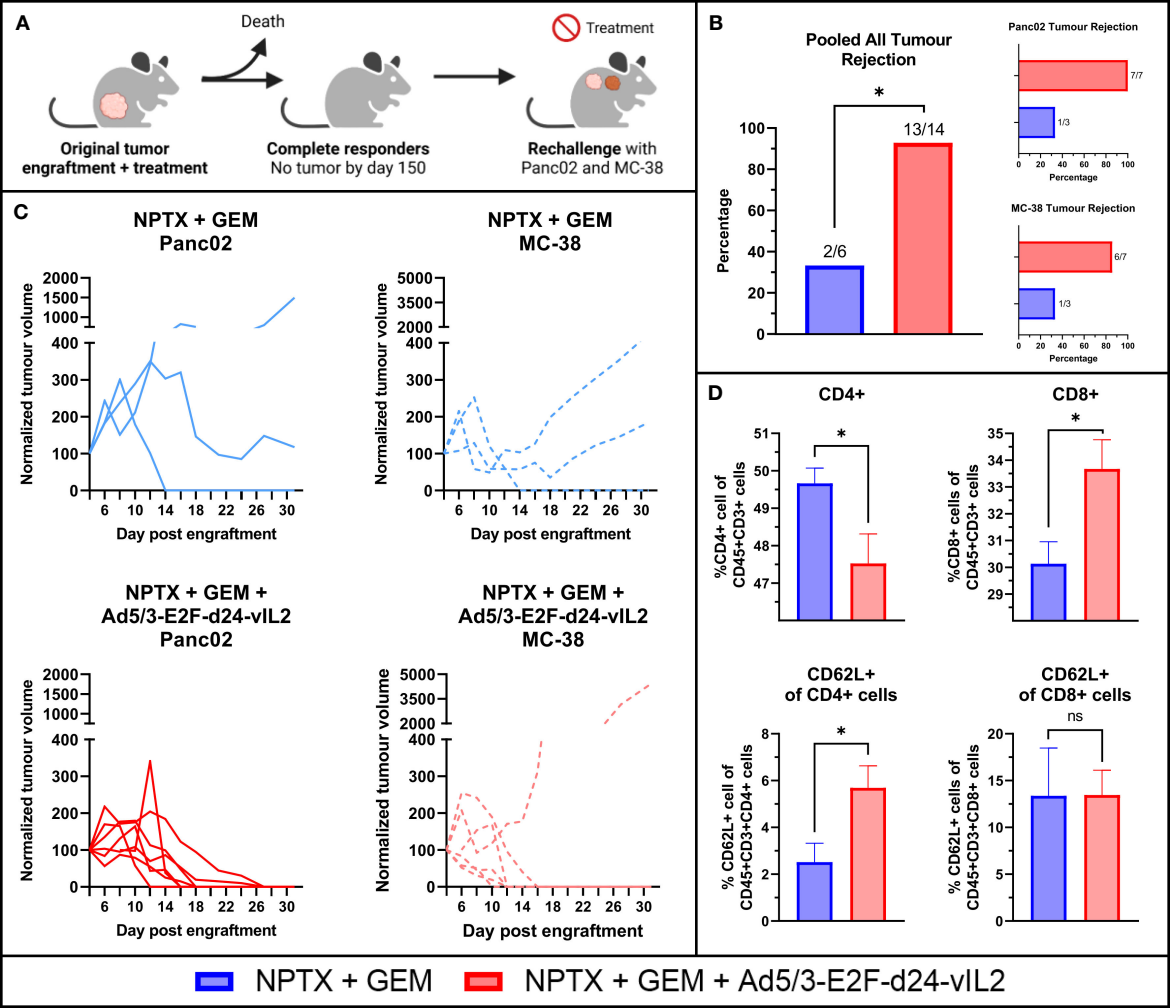
Re-challenge animal experiment. **(A)** Rechallenge animal experiment plan. **(B)** Pooled all tumor rejection, rejection of original Panc02 and rejection of un-encountered MC-38. **(C)** Individual tumor growth curves **(D)** Evaluation of CD4+, CD8+ splenocytes and their CD62L positivity evaluating memory formation in splenocytes after 31 days. N=7 for NPTX + GEM + vIL2 group, N=3 for NPTX + GEM. Difference in tumor rejection measured with Fisher’s exact test. Difference in immune cells between groups measured by an unpaired T-test with Welch’s correction. Error presented as SEM. Significance denoted as p-value < 0.05 = *.

Animals that were alive and showing complete tumor regression on day 150 were utilized for a re-challenge experiment. A total of 7 animals from the combination therapy group, 3 animals in the chemotherapy monotherapy group and 1 animal in the virus monotherapy group were eligible for the re-challenge experiment. These animals were re-challenged with the original cell line Panc02 and a previously un-encountered cell line MC-38, a murine colon cancer cell line. Tumor cells were engrafted to the upper back of the animals, after which tumor growth was measured and animals received no treatments (**Figure 7A**). Animals in the combination therapy group rejected the original Panc02 cells in all cases, whereas animals in the chemotherapy monotherapy group grew tumors in all except in one case (**Figure 7B**). Individual tumor growth curves are shown in **Figure 7C** . Interestingly, animals in the combination therapy group also showed high rejection of MC-38 cells, with all but one animal showing rejection. In total, the combination therapy group rejected all but one tumor, showing statistically significantly better protection (p=0.013).

After maximum tumor size was reached in one animal, all animals were euthanized at the same time and tumors (if present) and spleens were collected. Flow cytometric analysis of the spleens showed that combination therapy resulted in higher proportions of CD8+ T cells and lower proportions of CD4+ cells (**Figure 7D**). Additionally, increased proportions of CD62L+ CD4+ T cells were seen in the combination therapy spleens, with no changes seen in CD8+CD62L+ cells.

## 4 Discussion

PDAC immunotherapy with checkpoint inhibitors or adaptive cell therapies has shown mostly poor results, due to the highly immunosuppressive TME and dense stroma typically found in tumors, leading ultimately to poor penetration of treatment agents (7–9, 22). Thus far, no immunotherapeutic drugs are approved for PDAC, except checkpoint inhibitors for microsatellite instability high (MSI-H) tumors, which represent less than 1% of all PDAC tumors (6).

In the present study, we show that Ad5/3-E2F-d24-vIL2 efficiently lysed PDAC cells *in vitro* and synergized with nab-paclitaxel and gemcitabine chemotherapeutic regimen in Panc02 mouse model. The combination of oncolytic adenoviruses with paclitaxel therapy has been studied previously in other malignancies than PDAC, suggesting increased virus production and immunogenic cell death (24–26). Additionally, studies have shown the potential of E1B-19K deletion harboring oncolytic adenoviruses to increase gemcitabine-induced cell death, a phenomena we also confirmed in the present study (27). Most importantly, the combination of chemotherapy to virotherapy did not hinder the virus’ ability to kill cancer cells, even at high chemotherapy concentrations.

Regarding paclitaxel, we identified clues to the molecular mechanism behind the response by the observation of mitotic slippage. Mitotic slippage is a known phenomenon of microtubule targeting drugs, but its relevance in combination treatments is not well understood. Previous research conducted in ovarian cancer has noted similar effects of mitotic slippage when using paclitaxel and oncolytic viruses, albeit with different types of oncolytic adenoviruses (28). We also observed higher amount of immunogenic cell death when combining chemotherapy to virus therapy. Immunogenic cell death is a well-known effect of oncolytic adenoviruses, and previous work has noted this phenomenon in soft-tissue sarcoma, lung cancer and ovarian cancer (29–32).

Pre-clinical oncolytic adenovirus research is challenged by the limitations of models, since human oncolytic adenoviruses replicate poorly in mouse cells (17, 18). Thus, a popular choice of model organism in oncolytic adenovirus research is the Syrian hamster (Mesocricetus auratus), which is semi permissive for human oncolytic adenovirus replication (33). On the other hand, usage of the Syrian hamster model is hampered by the limited amount of research reagents, even though now more reagents are being developed also for immuno-oncology research (34). Immunocompromised mouse models are also a popular choice of model organism, but these models often have artificial immune systems and other limitations, such as graft-versus-host-disease. Therefore, in order to study the immunological effects of our therapy in an unmodified immune system, we chose the immunocompetent C57BL/6 as our model, with the sacrifice of oncolytic property of the virus but retaining the effects of the transgene and eventual anti-viral and anti-tumor immune response. This model allows the immunological study of the treatment, but it underestimates the overall efficacy of the virus. Nevertheless, better tumor growth control and survival were seen when virus monotherapy was compared to mock.

Characterization of *in vivo* materials demonstrated activation of immune cells and reprogramming of the TME. When comparing the chemotherapy group to the combination group, a decrease in markers of immunosuppression was seen, with increase in cell cytotoxicity markers. Importantly, no increase in the proportion of T regs were seen after therapy, showing that Ad5/3-E2F-d24-vIL2 is able to synergize with chemotherapy without increasing T reg percentages in tumors. Interestingly, the virus monotherapy group showed similar changes in the immune cell compartments as the combination therapy group, but the latter had better survival and tumor control. This effect is most likely explained by limitations of the model: no virus mediated oncolysis is present and thus the cytotoxic effect of chemotherapy plays a large role in tumor debulking, although not activating the host anti-viral response. In a replication permissive model, we previously showed vIL-2 virus monotherapy able to cure 62.5% of the animals in a hamster PDAC model (12).

Furthermore, the group receiving the combination therapy showed smallest amount of IL-6 and VEGF, which are known mediators of cancer cell growth and tumor angiogenesis, and associate with poor prognosis in many cancer types (35–37). Similar findings in downregulation of IL-6 have been noted with other oncolytic adenoviruses coding for immunostimulatory transgenes in different tumor models, indicating their ability to transform the immunosuppressive environment of tumors (38, 39). In PDAC, IL-6 is a key trans-signaling molecule, originally secreted from prelesions and leading to a vicious cycle of IL-6 excretion from other cell types of the TME, such as myeloid cells and CAFs, ultimately promoting cancer cell growth and immunosuppression (40, 41).

These findings are intriguing considering that CAFs are key stromal players in cancer, and intensive research is conducted to target CAFs for treatment (42). Direct depletion of CAFs *in vivo* led to increased metastasis and diminished survival, suggesting CAFs are not a homogenous population of immunosuppressive cells, hence the modulation of the CAF compartment *via* different avenues is favorable (43). In this research project, we showed that Ad5/3-E2F-d24-vIL2 is able to decrease the proportion of inflammatory CAFs and increase the proportion of antigen presenting CAFs in the tumors. This work builds on our previous research with Ad5/3-E2F-d24-vIL2, which showed that the effects are mediated by downregulation of immunosuppressive myeloid cells (12).

Furthermore, our findings in the re-challenge model underline a possibly important feature of oncolytic adenoviruses. Protection from re-challenge to the original cancer cell line was observed in all animals that received virotherapy, and more strikingly, protection was also observed against a previously non-encountered cell line. We have noted similar effects with different but related oncolytic adenoviruses in other models, including melanoma and hamster model of PDAC (12, 14, 44, 45). This effect is most likely caused by epitope spreading, leading to increased T cell clonality, which apparently occurs even in the absence of active oncolysis (43).

In conclusion, our study presents the feasibility of Ad5/3-E2F-d24-vIL2 as a immunotherapeutic regimen in Panc02 mouse model in combination with nab-paclitaxel and gemcitabine. Animals treated with the combination showed benefit in survival and tumor control. Further research in the topic is needed to fully decipher especially the effects of adenovirus treatment on the myeloid compartment of tumors and the possibility of further therapeutic combinations.

## Data availability statement

The original contributions presented in the study are included in the article/**Supplementary Material**. Further inquiries can be directed to the corresponding author.

## Ethics statement

The animal study was reviewed and approved by Animal Experimentation Board of the Provincial Government of Southern Finland (license number ESAVI/12559/2021).

## Author contributions

SP, DQ, JS, VC-C, RH, and AH designed the experiments. SP, DQ, TK, JC, SG-V-K, CH, SB, EJ, VA, LH, and MA performed the experiments. SP, DQ, TK, JC, SG-V-K, SB, EJ, VA, LH, CH, JS, VC-C, RH, MA, and AH analyzed the results. All authors were involved with writing and critical revision of the manuscript. All authors contributed to the article and approved the submitted version.

## Supplementary material

Supplementary Table 1 Antibodies used in flow cytometric assays. BL = Biolegend, BD = BD Biosciences, Thermo = Thermo Fischer Scientific.

Supplementary Figure 1 Flow cytometric gating strategy for cell cycle analysis. Tumor cells were identified with SSC-H and FSC-H. Doublet exclusion was performed with FSC-A to FSC-H and SSC-A to SSC-H gating. Ap = apoptosis, M = mitosis, MS = mitotic slippage.

Supplementary Figure 2 Classical cell cycle histograms for each replicate for **(A)** 24 hours post treatment and **(B)** 48 hours post treatment.

Supplementary Figure 3
**(A)** Evaluation of different oncolytic adenovirus infectivity against Panc02 cell line. **(B)** Production of cytokine in Panc02 cell line.

Supplementary Table 2 Histopathological analysis of animal internal organs collected on Day 10 and analyzed by veterinary pathologist. NSL=no specific changes.

Supplementary Table 3 Absolute number of collected cells and cells collected per panel on Day 10 in relation to tumor volume on Day 10.
