# Supplementary figures and images for: An oncolytic adenovirus coding for a variant interleukin 2 cytokine improves response to chemotherapy through enhancement of effector lymphocyte cytotoxicity, fibroblast compartment modulation and mitotic slippage
Source: Front Immunol. 2023 Jul 5;14:1171083. doi: 10.3389/fimmu.2023.1171083 (PMC10354511; doi:10.3389/fimmu.2023.1171083)

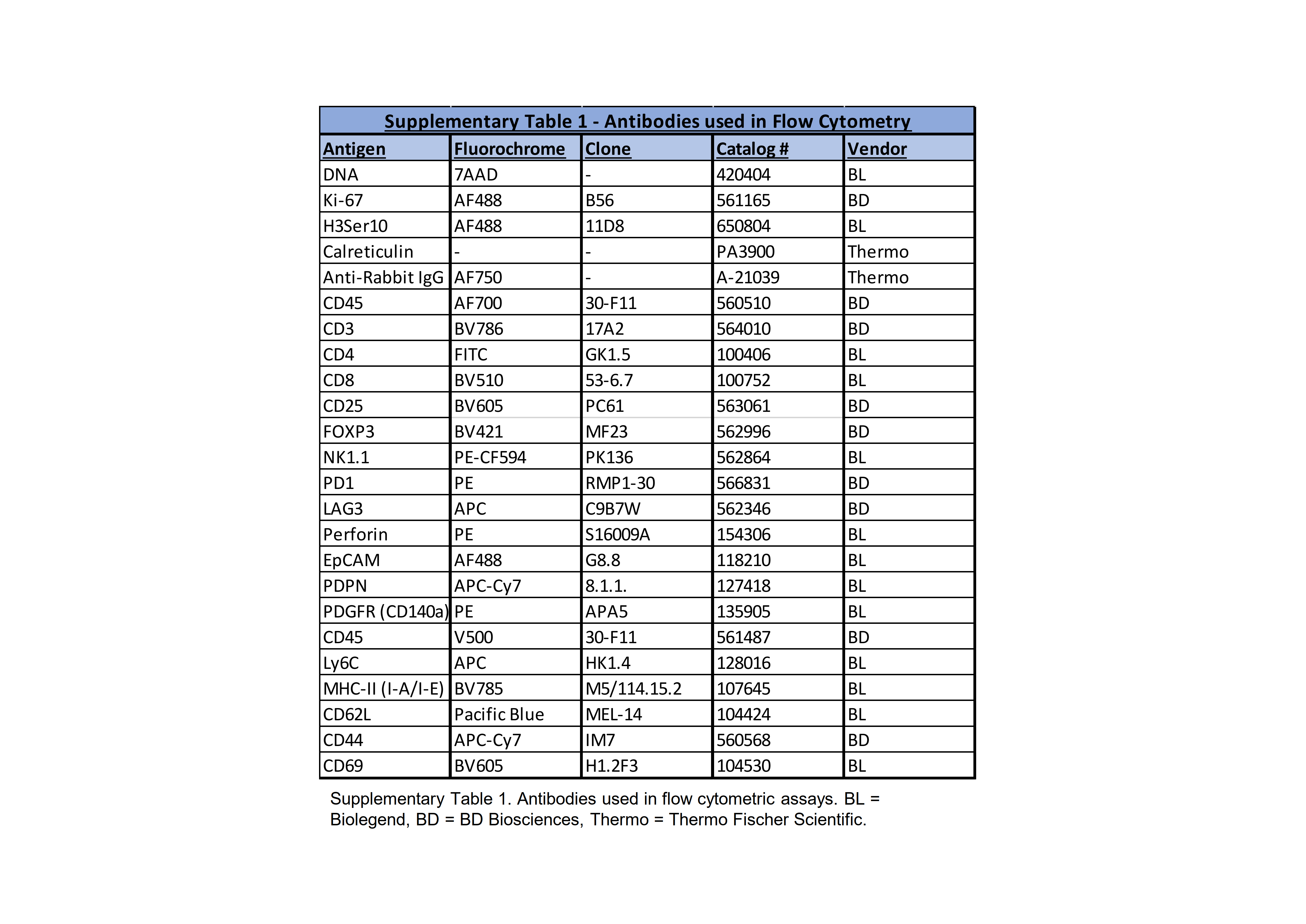

Supplement: Supplementary Table 1 — Antibodies used in flow cytometric assays. BL = Biolegend, BD = BD Biosciences, Thermo = Thermo Fischer Scientific. [file Image_1.tif]

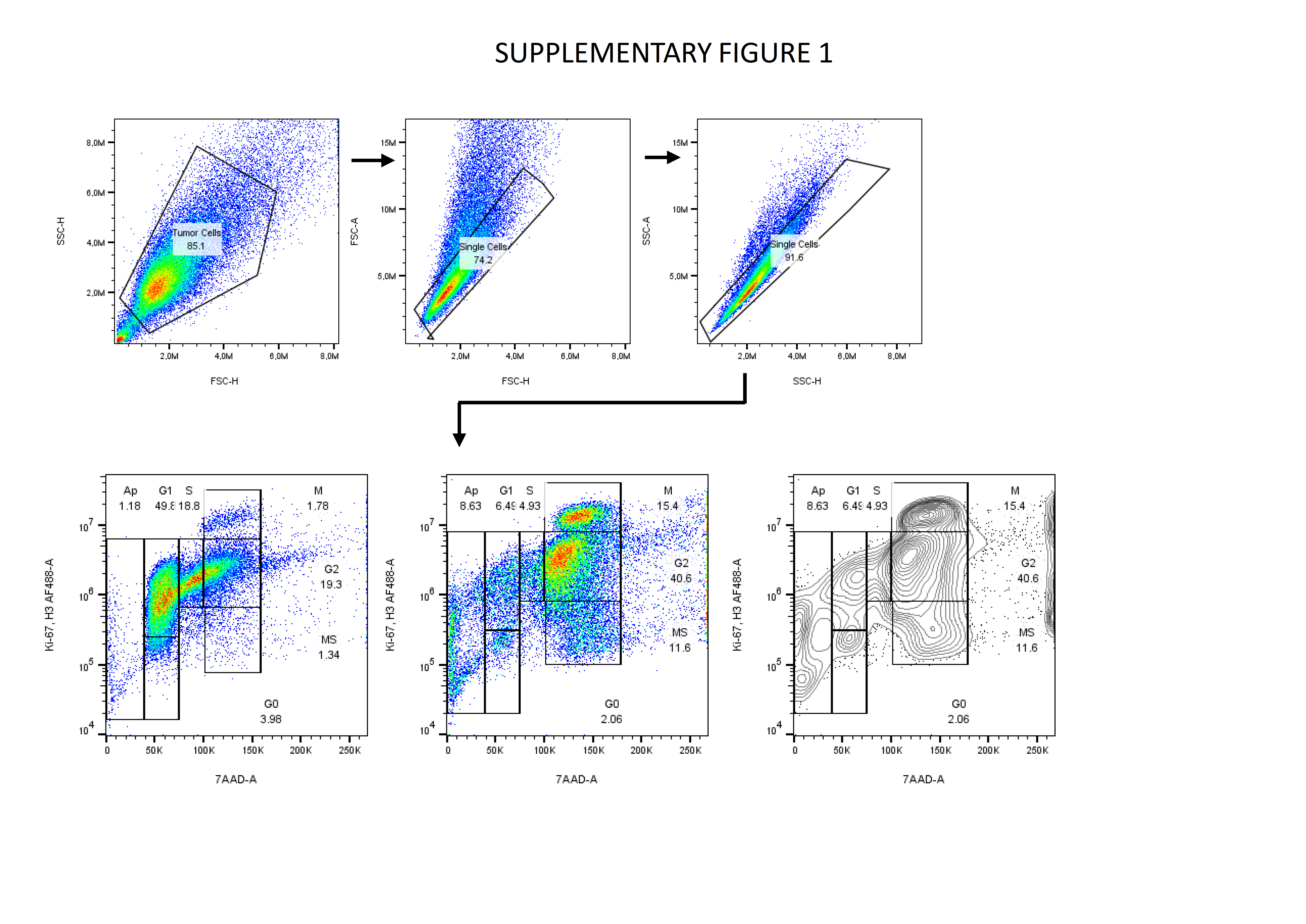

Supplement: Supplementary Figure 1 — Flow cytometric gating strategy for cell cycle analysis. Tumor cells were identified with SSC-H and FSC-H. Doublet exclusion was performed with FSC-A to FSC-H and SSC-A to SSC-H gating. Ap = apoptosis, M = mitosis, MS = mitotic slippage. [file Image_2.tif]

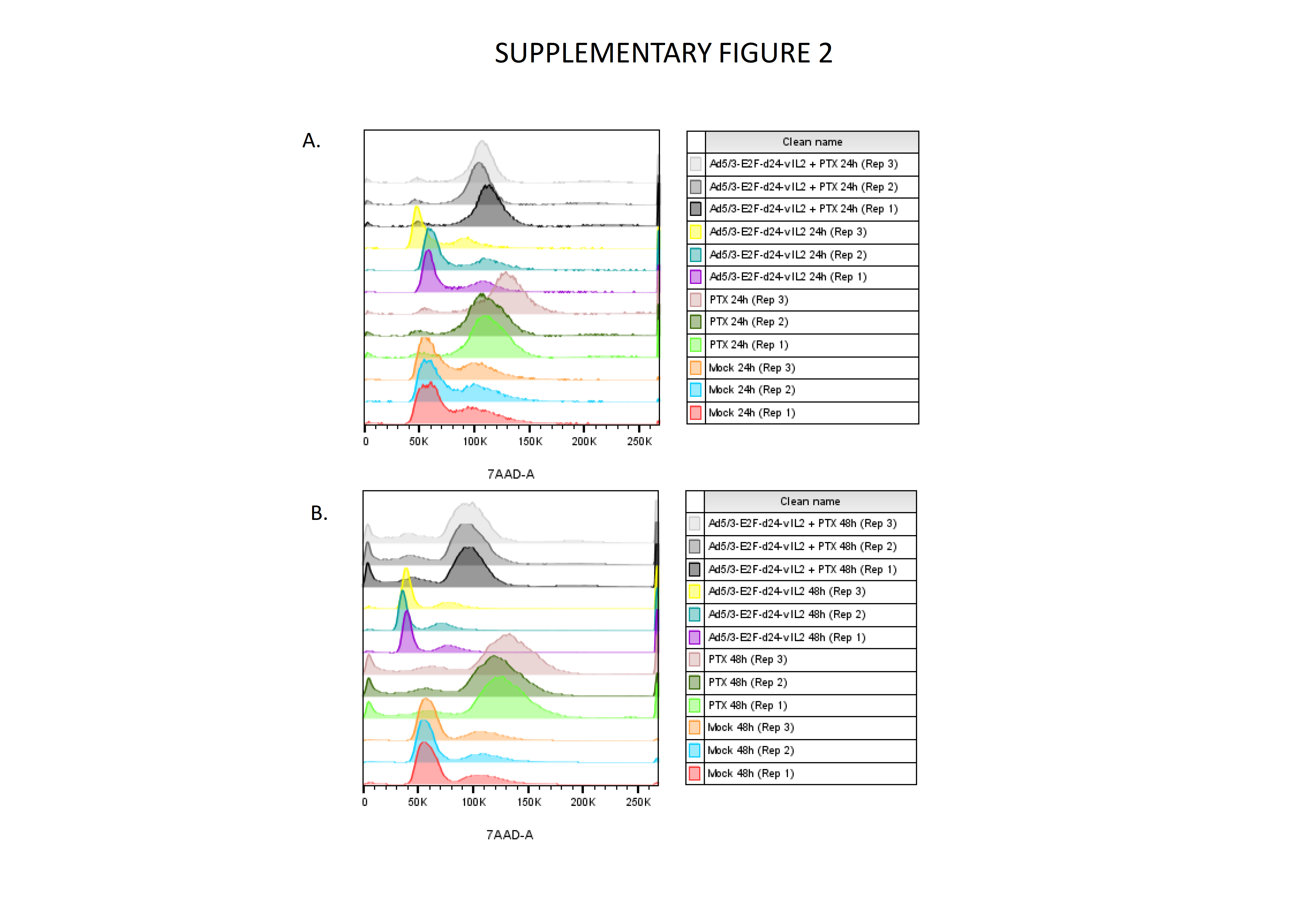

Supplement: Supplementary Figure 2 — Classical cell cycle histograms for each replicate for (A) 24 hours post treatment and (B) 48 hours post treatment. [file Image_3.tif]

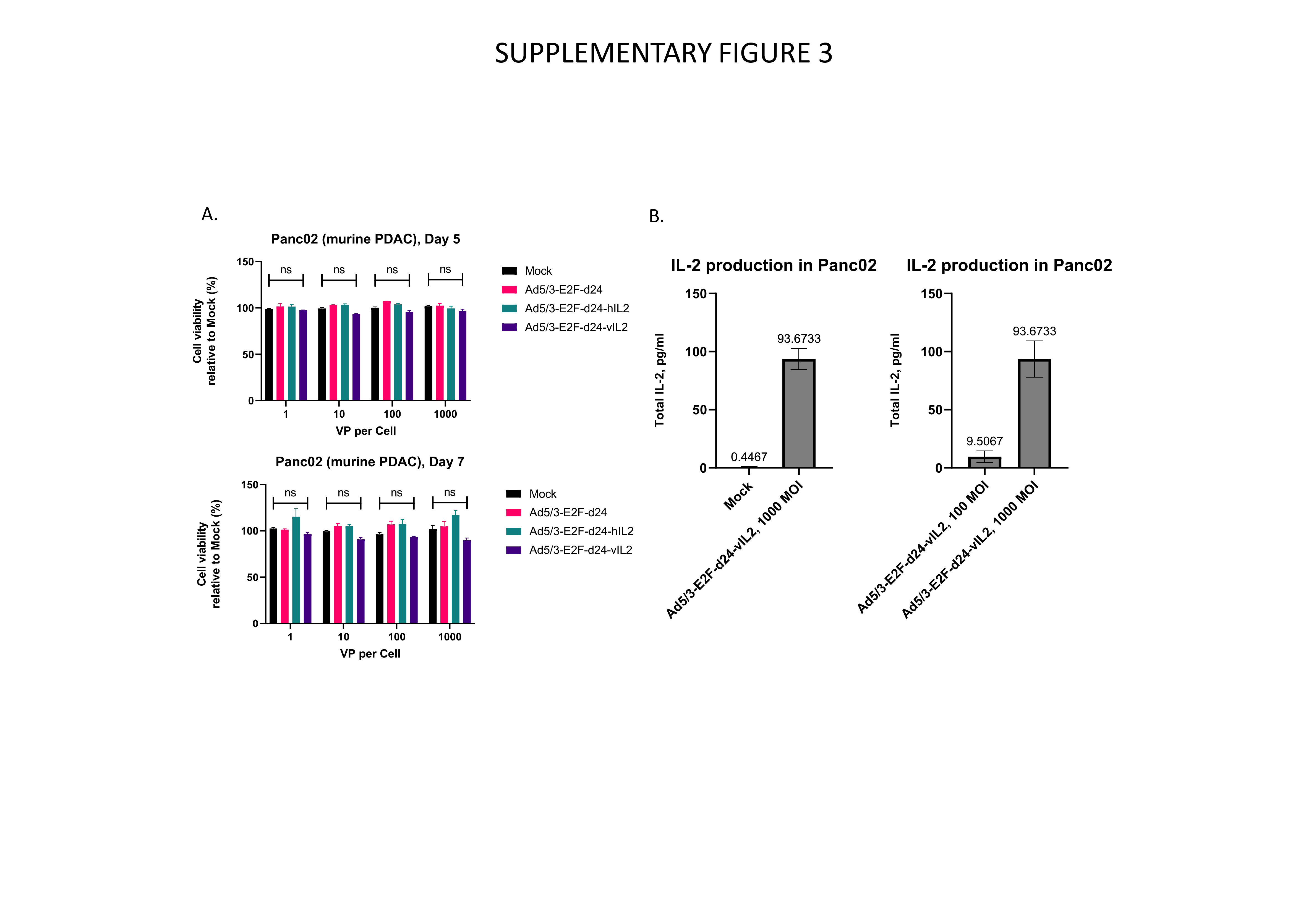

Supplement: Supplementary Figure 3 — (A) Evaluation of different oncolytic adenovirus infectivity against Panc02 cell line. (B) Production of cytokine in Panc02 cell line. [file Image_4.tif]

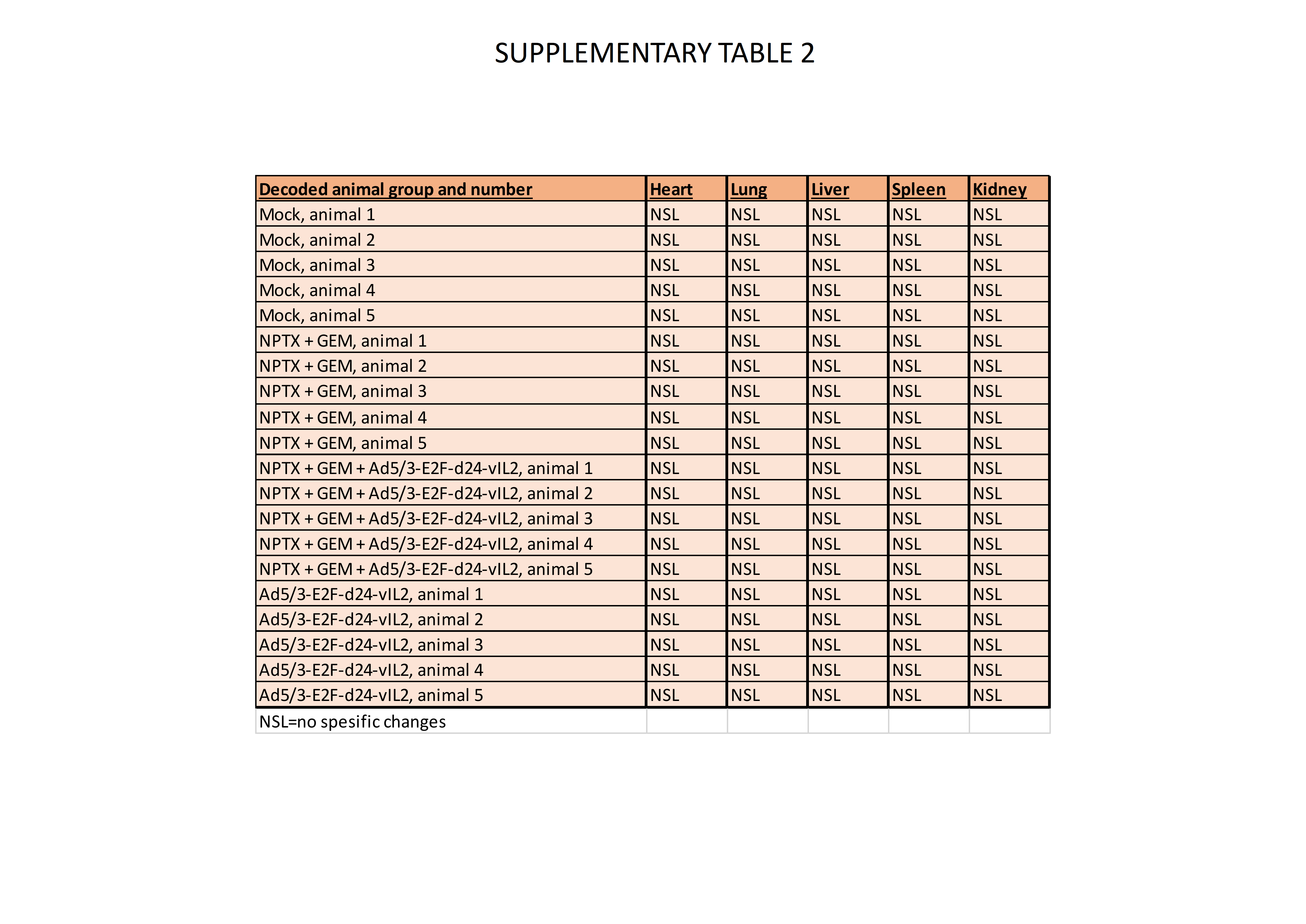

Supplement: Supplementary Table 2 — Histopathological analysis of animal internal organs collected on Day 10 and analyzed by veterinary pathologist. NSL=no specific changes. [file Image_5.tif]

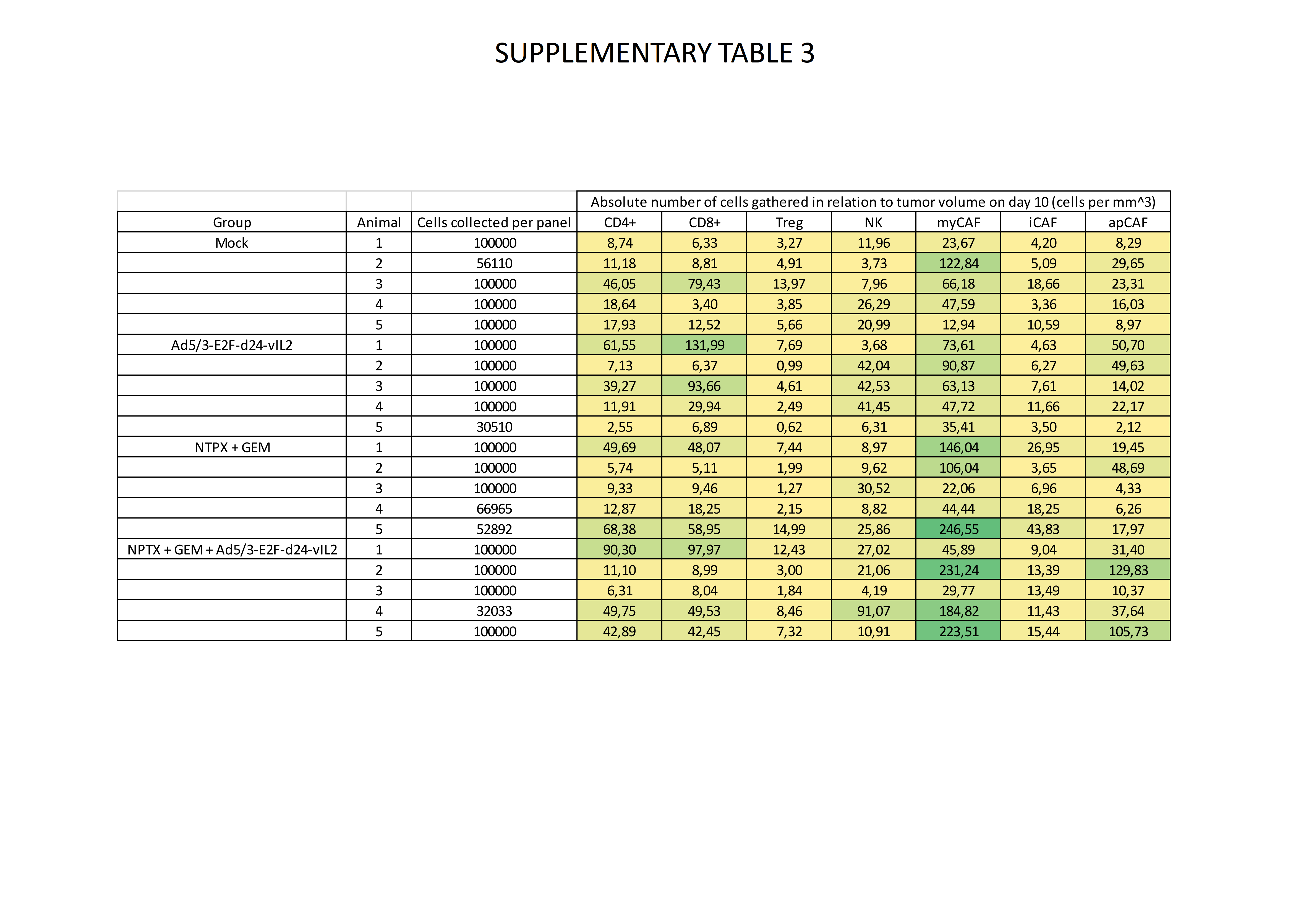

Supplement: Supplementary Table 3 — Absolute number of collected cells and cells collected per panel on Day 10 in relation to tumor volume on Day 10. [file Image_6.tif]
